# Patient‐reported factors associated with early arrival for stroke treatment

**DOI:** 10.1002/brb3.2225

**Published:** 2021-06-04

**Authors:** Heidi S. Eddelien, Jawad H. Butt, André C. Amtoft, Nicholine S. K. Nielsen, Emilie S. Jensen, Ida M. K. Danielsen, Thomas Christensen, Anne K. Danielsen, Nete Hornnes, Christina Kruuse

**Affiliations:** ^1^ Faculty of Health and Medical Sciences Institute of Clinical Medicine University of Copenhagen Copenhagen Denmark; ^2^ Neurovascular Research Unit Herlev Gentofte Hospital University of Copenhagen Copenhagen Denmark; ^3^ Department of Cardiology Rigshospitalet University of Copenhagen Copenhagen Denmark; ^4^ Department of Neurology Nordsjællands Hospital University of Copenhagen Copenhagen Denmark; ^5^ Department of Gastroenterology Herlev Gentofte Hospital University of Copenhagen Copenhagen Denmark; ^6^ Department of Neurology Herlev Gentofte Hospital University of Copenhagen Copenhagen Denmark

**Keywords:** prehospital emergency care, stroke knowledge, stroke

## Abstract

**Objective:**

Timely evaluation and initiation of treatment is the key for improving stroke outcomes, although minimizing the time from symptom onset to the first contact with healthcare professionals remains a challenge. We aimed to identify patient‐related factors associated with early hospital arrival.

**Materials and methods:**

In this cross‐sectional survey, we included patients with stroke or transient ischemic attack admitted directly to one of two noncomprehensive stroke units or transferred to the units from comprehensive stroke centers in the Capital Region of Denmark. Patient‐reported factors associated with early hospital arrival were analyzed using multivariable logistic regression analysis adjusted for age, sex, education, living arrangement, brain location of the stroke, stroke severity, patient‐perceived symptom severity, history of prior stroke, stroke risk factors, and knowledge of stroke symptoms.

**Results:**

In total, 479 patients with acute stroke were included (median age 74 (25th–75th percentile, 64–80), 40% women), of whom 46.4% arrived within 180 min of symptom onset. Factors associated with early hospital arrival were patients or bystanders choosing emergency medical service (EMS) for the first contact with a medical professional (adjusted odds ratio (OR), 3.41; 95% confidence interval, CI [1.57, 7.35]) or the patient's perceived symptom severity above the median score of 25 on a 100‐point verbal scale (adjusted OR, 2.44; 95% CI [1.57, 3.82]). Living alone reduced the likelihood of early arrival (adjusted OR, 0.53; 95% CI [0.33, 0.86]).

**Conclusions:**

Only when patients perceived symptoms as severe or when EMS was selected as the first contact, early arrival for stroke treatment was ensured.

## Graphical abstract

Patient‐related factors associated with hospital arrival within 180 minutes

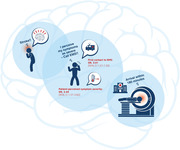

## 1 INTRODUCTION

Although the overall door‐to‐treatment time for acute stroke has improved greatly, shortening the time from symptom onset to making the first contact with healthcare professionals is of specific interest (Beckett et al., 2015; Fassbender et al., 2013; Mackintosh et al., 2012; Mellor et al., 2015). To enable the “fast tracking” of in‐hospital treatment, a major focus for overcoming challenges to rapid prehospital response has substantially reduced the time from the first contact to hospital arrival (Simonsen et al., 2014). However, less attention has been directed toward the causes of and barriers to patients seeking immediate help (Fassbender et al., 2013, p. 6). Minimizing patient delay when seeking medical care is crucial during an ischemic stroke, as increased time until treatment with intravenous thrombolysis using recombinant tissue type plasminogen activator or endovascular treatment is associated with worse outcomes. In contrast, early admission to dedicated stroke units improves outcomes of acute ischemic stroke (Emberson et al., 2014; Goyal et al., 2019; Lees et al., 2016; Powers et al., 2018; Saver et al., 2013; Whiteley et al., 2016). In addition to rapid treatment of ischemic stroke, spontaneous intracerebral hemorrhage requires early intensive care to lower blood pressure, promptly reversal of the anticoagulant, allow for surgical intervention when needed, and transfer to an intensive care unit or dedicated stroke unit to improve the outcome (Cordonnier et al., 2018; Hemphill et al., 2015). Globally, only a minor proportion of eligible patients receive revascularization therapy, in part due to delays in patients’ responses to symptom onset (Fassbender et al., 2013). Stroke sequelae impose a personal cost for patients and their families and a substantial socioeconomic impact to the public health burden (Cordonnier et al., 2018; Fassbender et al., 2013; Johnson et al., 2019; Lackland et al., 2014). To shorten the time from symptom onset to hospital arrival for stroke patients, we must identify patient‐related factors that impact this time frame. In this cross‐sectional, two‐center study, we aimed to distinguish such factors associated with the first contact to the healthcare system and subsequent hospital arrival, particularly within 180 min from the onset of stroke symptoms.

## 2 MATERIALS AND METHODS

### 2.1 Study design and setting

We performed a cross‐sectional survey at two noncomprehensive stroke centers in the Capital Region of Denmark. In Denmark, all citizens are assigned a general practitioner (GP), whom they can contact during normal business hours or an out‐of‐hours primary care service (OOH‐PC). If the medical condition requires immediate attention, all citizens should call emergency medical service (EMS). The structured questionnaire was designed specifically for this study. To verify the internal validity, development of the questionnaire included an assessment of face validity and content validity (Streiner and Norman, 2008). To assess item clarity, cognitive interviewing of 20 patients similar to the target population was performed before the questionnaire's general application. Challenging questions were reworded and then repeated using verbal probing until no cognitive issues were identified (Andersen et al., 2010).

### 2.2 Study population

Patients were eligible for enrollment if they fulfilled all of the following inclusion criteria: (1) stroke diagnosis (International Classification of Diseases (ICD) codes I61: nontraumatic intracerebral hemorrhage; I63: ischemic stroke (cerebral infarction); or G45: transient ischemic attack, TIA) was based on clinical examination by a neurologist and neuroimaging (CT and MRI scans), (2) admitted directly to a noncomprehensive stroke center or transferred from a comprehensive or primary stroke center after an evaluation for or treatment with thrombolysis and/or thrombectomy, (3) age ≥18, and (4) obtained written informed consent from the patient. If the patient could not contribute to information on route of admission or causes of delay due to, for example. aphasia, a bystander (the patients’ medical proxy who was on site during stroke onset) could contribute to information during interview with consent from the patient. Patients were excluded if they had (1) a subarachnoid hemorrhage, (2) an in‐hospital stroke, (3) a nonstroke diagnosis, or (4) symptoms that began when the patient was abroad. Only the first event was included in the analysis.

### 2.3 Data collection

Data were collected at Herlev Gentofte Hospital from February 2018 to June 2018 and at Nordsjællands Hospital from September 2018 to January 2019. Immediately after admission to the stroke unit but prior to diagnosis confirmation, patients were approached by a research assistant trained in administering the structured questionnaire. Medical charts and EMS data were used to supplement the patients’ questionnaire responses. Data were recorded and managed in an electronic case report system (Harris et al., 2009).

### 2.4 Variables

Time of stroke symptom onset was defined as the time when the patient or bystander first noticed stroke symptoms. Unknown stroke onset was defined as the time the patient was last seen well (Powers et al., 2018). If the exact time of stroke onset could not be specified, the time was estimated to morning (8:00), noon (12:00), afternoon (16:00), evening (20:00), and night (0:00). Time of the first contact to the healthcare system was defined as the time between symptom onset and the first call for medical assistance by the patient or the bystander. The exact time was retrieved from prehospital records or medical charts in the case of EMS contact. Early arrival was defined as the patients’ arrival at the first hospital within 180 min from symptom onset (Powers et al., 2018). Age was included as a continuous variable. Education level was categorized into basic, further, or higher. Living arrangement was dichotomized into living alone or with someone. Stroke severity was assessed using the Scandinavian Stroke Scale (SSS), which is mandatory in the Danish National Stroke Registry (Damgaard & Vögele, 2019), and classified as severe (0–25 points), moderate (26–42 points), and mild (43–58 points) (Govan et al., 2009). Stroke location in the brain was categorized into right hemisphere, left hemisphere, or bilateral, brainstem, and/or cerebellum. Relevant comorbidities were self‐reported and verified by medical records and included hypertension, diabetes, atrial fibrillation, hypercholesterolemia, prior acute myocardial infarction, claudication, carotid artery stenosis, heart failure, sleep apnea, and prior stroke. Smoking status (current, former, or never) was also recorded. The patient's use of prehospital medication for one or more stroke risk factors (hypertension, diabetes, atrial fibrillation, hypercholesterolemia, acute myocardial infarction, claudication, carotid artery stenosis, heart failure, prior stroke) was defined as a dichotomous variable. Patient‐reported typical stroke symptoms included facial drooping, arm or leg weakness, and speech difficulties. Patient‐perceived symptom severity was rated on a scale from 0 to 100, where 100 was most severe (Streiner and Norman, 2008). A perceived high level of symptom severity was pragmatically defined using the median as the cutoff. Stroke recognition was classified as by the patient or by others, the latter including health professionals, family, friends, neighbors, co‐workers, or bystander's unknown to the patient. Help‐seeking behavior included contacting EMS, health professionals (e.g., GP, other doctors, nurses), and a nonmedical personnel (family, friends, neighbors, co‐workers, bystanders). The patient's prior knowledge of acute stroke therapy was recorded “yes” or “no.”

### 2.5 Statistical methods

Statistical analyses were performed using SAS version 9.4 (SAS Institute, Cary, NC). A two‐sided significance level was set at alpha ˂.05. Continuous baseline characteristics are reported as medians with 25th and 75th percentiles. Differences between proportions were examined by chi‐square test or Fisher's exact test. To identify factors associated with early arrival, multivariable logistic regression models were used to estimate OR with 95% confidence interval (CI) adjusted for covariates (sex, age, level of education, living arrangement, type of the first contact, stroke location, SSS score, self‐perceived symptom severity, knowledge of acute stroke therapy, prehospital medication for stroke risk factors, and patient‐reported stroke symptoms). For sensitivity purposes, we examined patient‐reported factors associated with time from symptom onset to hospitalization within 270 min (Emberson et al., 2014; Saver et al., 2013). We also examined the time from symptom onset to the first contact of any type within 270 min. The primary outcome was further examined in an additional analysis, which only included results from interviews performed with the patient alone (370 out of 479) to identify how bystanders’ responses may influence results.

### 2.6 Ethics

The study was approved by the Capital Region Ethics Committee (no. 2012‐58‐004) and the Danish Data Protection Agency (no. 2012‐58‐0004; internal reference: HGH‐2017‐110, I‐Suite no. 06014). Patients provided written informed consent before study enrollment.

### 2.7 Availability of data

The data analyzed in this study are available from the corresponding author upon reasonable request and in adherence to Danish legislation.

## 3 RESULTS

### 3.1 Patient characteristic

**FIGURE 1:**
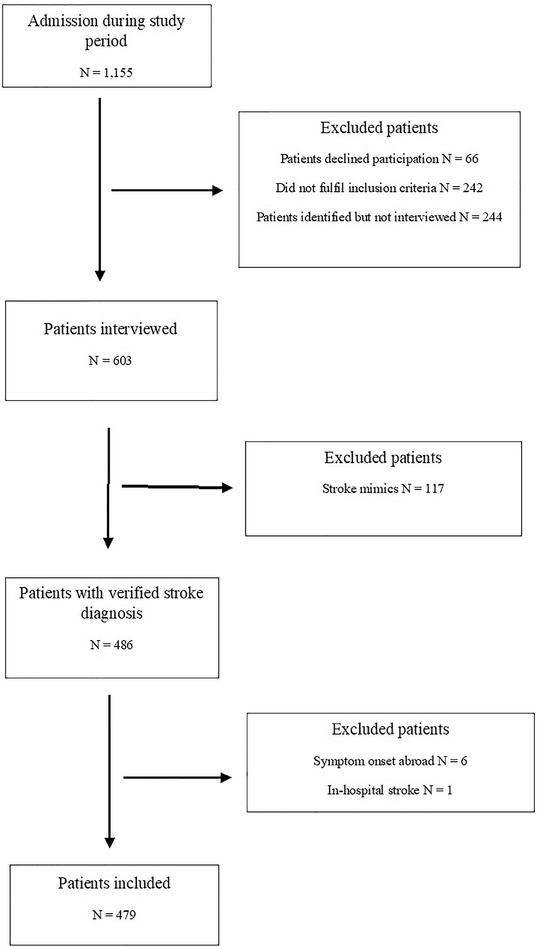
Patient enrollment

Figure 1 summarizes the patient enrollment process. A total of 1155 patients were screened during the inclusion periods. Of these, 66 patients declined participation, 242 patients did not meet inclusion criteria, and 244 patients were identified but not approached due to time restrictions. A total of 603 patients were eligible and consented to participate in the interview. Of these, 124 patients were excluded following final clinical workup. Thus, the total study population comprised 479 patients. A total of 370 interviews were performed with the patient alone, 86 interviews included both the patient and bystander, and 23 interviews were performed with the bystander only. The median age of the study population was 74 (64–80), and 40% were females. Stroke subcategory was distributed as follows: nontraumatic intracerebral hemorrhage (8.2%,) ischemic stroke (64.5%), and TIA (27.3%). Stroke was recognized by the patient in 84.1% of cases and by others in 17.1 % of cases. The demographic characteristics are presented in Table 1.

**TABLE 1:** Patient characteristics and time from symptom onset to hospital arrival

	˂180 min	>180 min	
Variables	*N* = 222 (46.35%)	*N* = 257 (53.65%)	p‐Value
Demographics			
‐ Age in years, ,median 25th−75th percentile	73 (64−79)	74 (64−81)	.93
‐ Female	88 (39.64)	104 (40.47)	.85
Level of education			.57
‐ Basic	84 (37.84)	106 (41.25)	
‐ Further	101 (45.50)	104 (40.47)	
‐ Higher	37 (16.67)	46 (17.90)	
‐ Not available	0 (0.00)	1 (0.39)	
Living arrangements			.63
‐ Living with someone	137 (61.71)	153 (59.53)	
‐ Living alone	85 (38.29)	104 (40.47)	
Scandinavian Stroke Scale score^a^			.0005
‐ Mild	174 (78.38)	323 (90.27)	
‐ Moderate	37 (16.67)	15 (5.84)	
‐ Severe	11 (4.95)	10 (3.89)	
Type of stroke			.04
‐ I61: Nontraumatic intracerebral hemorrhage	18 (8.11)	21 (8.17)	
‐ I63: Cerebral infarction	131 (59.01)	178 (69.26)	
‐ G45: Transient cerebral ischemic attacks	73 (32.88)	58 (22.57)	
Stroke location			.51
‐ Left hemisphere	91 (40.99)	97 (37.74)	
‐ Right hemisphere	79 (35.59)	88 (34.24)	
‐ Bilateral, brainstem, cerebellum	52 (23.42)	72 (28.02)	
Risk factors, history of			
‐ Hypertension	124 (55.86)	140 (54.47)	.76
‐ Diabetes	23 (10.36)	31 (12.06)	.56
‐ Atrial fibrillation	50 (22.52)	53 (20.62)	.61
‐ Hypercholesterolemia	101 (45.50)	116 (45.14)	.94
‐ Acute myocardial infarct	17 (7.66)	19 (7.39)	.91
‐ Claudication	23 (10.36)	17 (6.61)	.14
‐ Carotid stenosis	16 (7.21)	20 (7.78)	.81
‐ Heart failure	20 (9.01)	17 (6.61)	.33
‐ Sleep apnea	10 (4.50)	26 (10.12)	.02
‐ Prior stroke	51 (22.97)	43 (16.73)	.09
‐ Smoking	46 (20.72)	61 (23.74)	.08
‐ Current	87 (39.19)	118 (45.91)	
‐ Former	89 (40.09)	78 (30.35)	
‐ Never			
Prehospital medication for stroke comorbidity			.122
‐ ≥1	149 (67.12)	155 (60.31)	
‐ None	73 (32.88)	102 (39.69)	
Typical acute stroke symptoms FAST			.56
‐ ≥1	113 (50.90)	124 (48.25)	
‐ None	109 (49.10)	133 (51.75)	
Typical acute stroke symptoms BEFAST			.73
‐ ≥1	114 (51.35)	128 (49.81)	
‐ None	108 (48.65)	129 (50.19)	
Perceived severity of symptoms^b^			.002
‐ ˂Median 25	90 (40.54)	145 (56.42)	
‐ >Median 25	119 (53.60)	101 (39.30)	
‐ Not available	13 (5.86)	11 (4.28)	
Stroke recognition			.008
‐ Patient	180 (81.08)	233 (90.66)	
‐ Bystander	38 (17.12)	23 (8.95)	
‐ Not available	4 (1.80)	1 (0.39)	
Prior knowledge of acute stroke therapy			.29
‐ Yes	129 (58.11)	137 (53.31)	
‐ No	93 (41.89)	120 (46.69)	
Help seeking behavior, first contact			˂.0001
‐ Emergency medical services	100 (45.05)	47 (18.29)	
‐ OOH‐PC	57 (25.68)	64 (24.90)	
‐ General practitioner	37 (16.67)	103 (40.08)	
‐ Home care	6 (2.70)	5 (1.95)	
‐ Out‐patient clinic	4 (1.80)	12 (4.67)	
‐ Family, friend, neighbor, co‐workers	14 (6.31)	25 (9.73)	
‐ Unknown bystander	0 (0.00)	1 (0.39)	
‐ None	4 (1.80)	0 (0.00)	
Treatment			
‐ Thrombolysis	49 (22.07)	8 (3.11)	˂.0001
‐ Thrombectomy	10 (4.50)	2 (0.78)	˂.009

^a^Scandinavian Stroke Scale classified as severe (0–25 points), moderate (26–42 points), and mild (43–58 points).

^b^Patient‐perceived symptom severity was rated on a scale from 0 to 100, where 100 was most severe.

Abbreviations: BEFAST, balance, eye, face, arm, speech, time; FAST , face, arm, speech, time; GP, general practitioner; OOH‐PC , out‐of‐hours primary care; SSS , Scandinavian Stroke Scale.

### 3.2 Primary outcome

**FIGURE 2:**
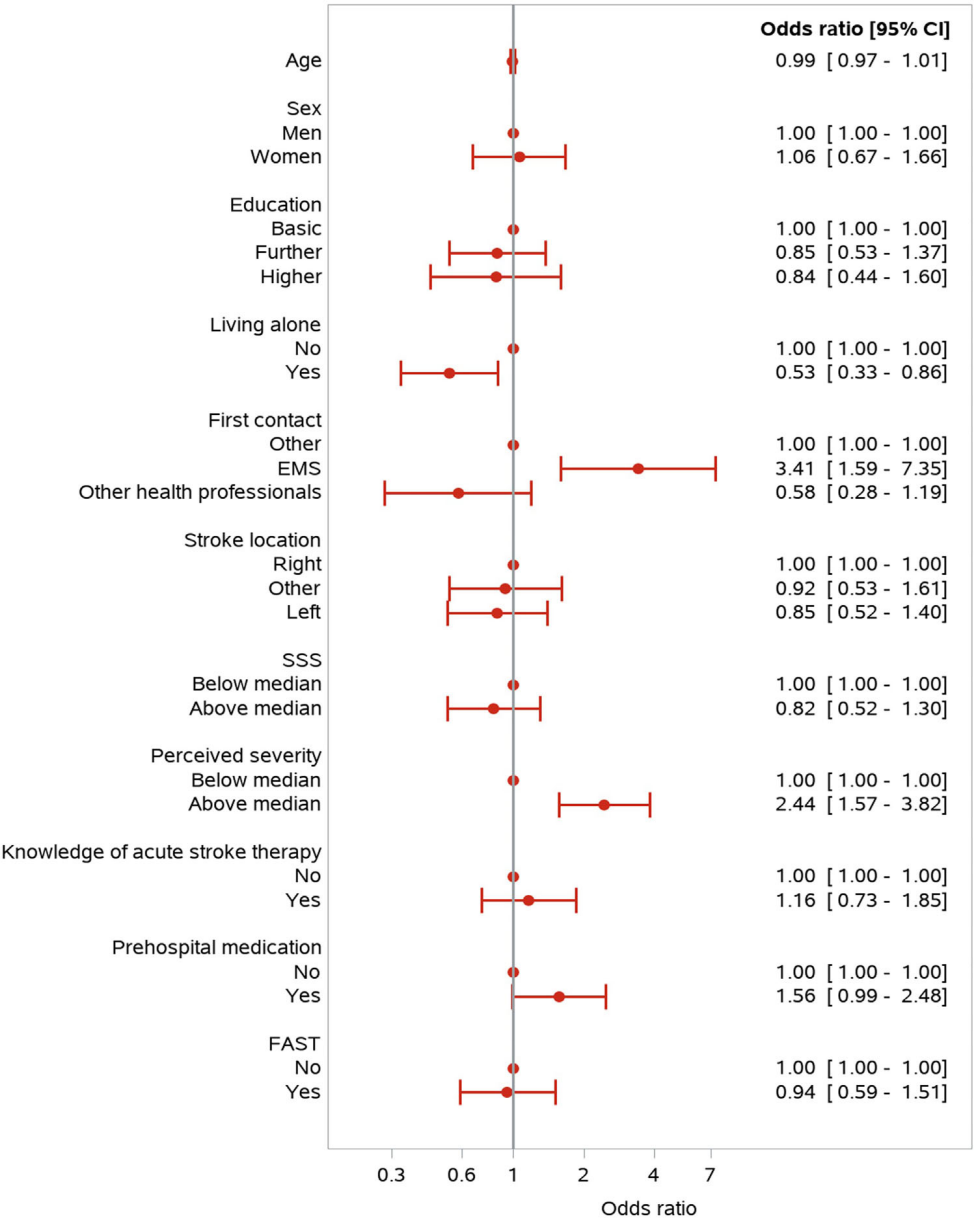
Symptom onset to hospital arrival <180 min *Note*: SSS, Scandinavian Stroke Scale score (median 56 out of 58; 58 no symptoms); FAST, face, arm, speech, time, which describes factors associated with the need to call EMS immediately for evaluation for treatment. Prehospital medication is related to stroke risk factors.

In total, 222 (46.3%) patients arrived to the hospital within 180 min. Of the 222 patients, 49 (22.1%) received recombinant tissue‐type plasminogen activator, and 10 patients (4.5%) underwent thrombectomy, which were a significantly higher proportion compared with those who arrived later than 180 min. Symptom severity was perceived as high in 119 patients (53.6%), 147 (60.4%) patients perceived that their situation required prompt action, and 85 (38.3%) lived alone (Table 1). The first contact to EMS and high patient‐perceived symptom severity were associated with hospital arrival within 180 min (adjusted OR, 3.41; 95% CI [1.59, 7.35]) and (adjusted OR, 2.44; 95% CI [1.57, 3.82]), respectively. However, living alone was associated with a lower likelihood of hospital arrival within 180 min (adjusted OR, 0.53; 95% CI [0.33, 0.86]) (Figure 2).

### 3.3 Secondary outcome

**FIGURE 3:**
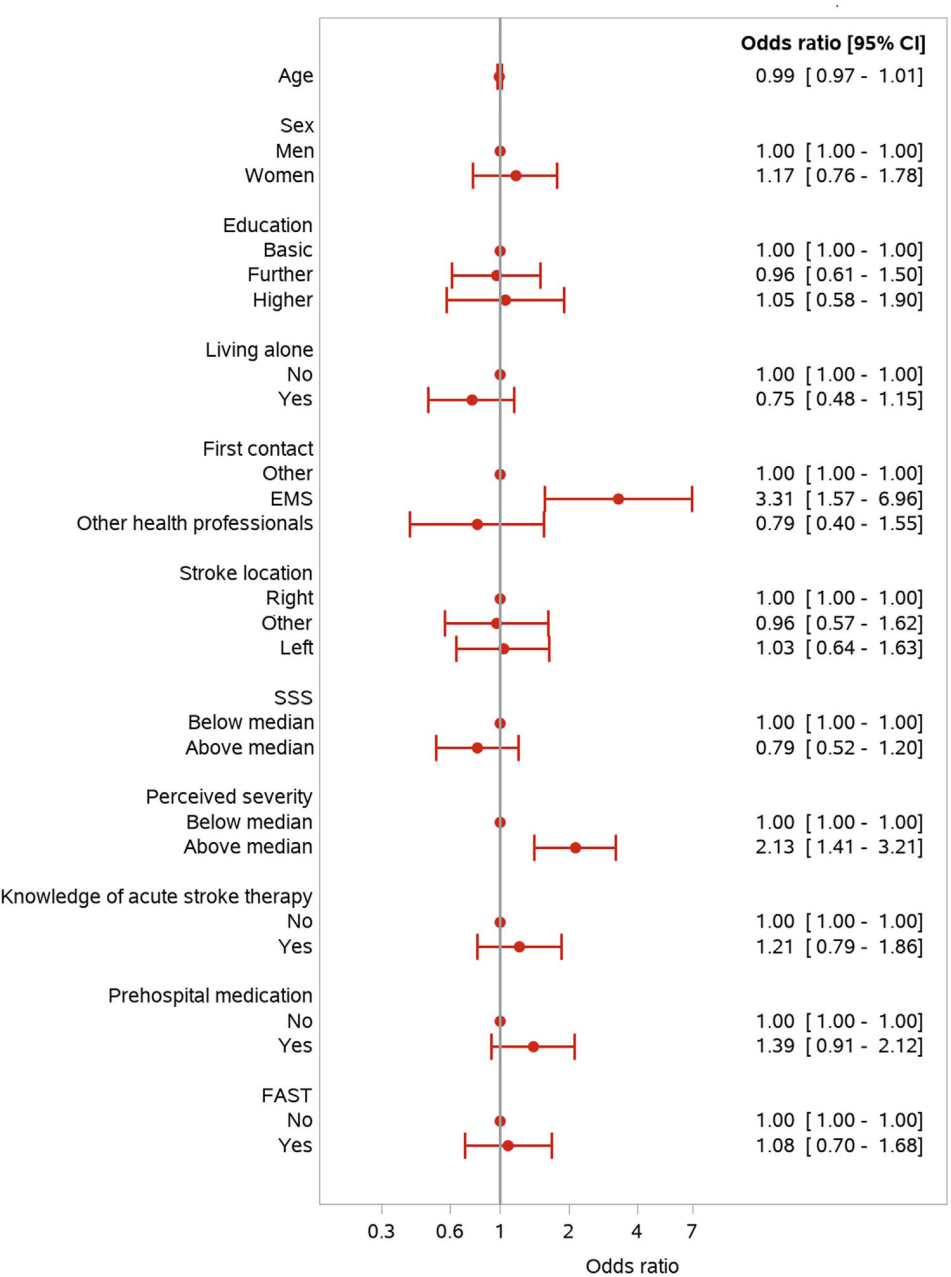
Symptom onset to the first contact <180 min *Note*: SSS, Scandinavian Stroke Scale score (median 56 out of 58; 58 no symptoms); FAST, face, arm, speech, time, which describes factors associated with the need to call EMS immediately for evaluation for treatment. Prehospital medication is related to stroke risk factors.

**FIGURE 4:**
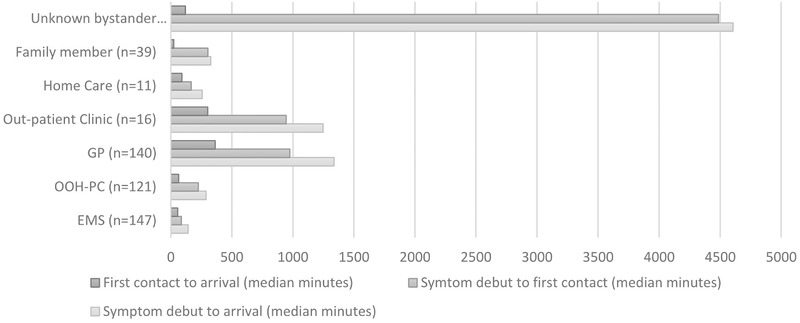
Time from stroke symptom onset to hospital arrival according to the first contact

Of the 222 patients who arrived within 180 min, EMS was the first choice of contact in hundred (45.1%) cases. EMS was also the first contact in 135 (31.2%) of patients having at least one typical stroke symptom but was the first contact in only 12 (25.5%) cases without a typical symptom. The median time from symptom onset to the first contact with EMS was 86 min (range: 24–270), and the median time from symptom onset to hospital arrival using EMS was 142 min (range: 68–332). Factors associated with the first contact within 180 min were the same as those for hospital arrival within 180 min (Figure 3). In contrast, the patient's GP was the first choice of contact in 37 (16.7%) cases, and the median time from symptom onset to the first contact was 975 min (149.5–3091). The median time from symptom onset to hospital arrival via GP was 1,337 min (range: 336–4217.5) (Figure 4). The longest time delay occurred between symptom onset and the decision to seek medical help (Figure 4).

### 3.4 Sensitivity analyses

To test the robustness of our findings, we also investigated time from symptom onset to hospital arrival within 270 min (Table I in the Supporting Information). Similar to the main analysis, calling EMS as the first contact after symptom onset and high patient‐perceived symptom severity were associated with hospital arrival within 270 min (adjusted OR, 3.13; 95% CI [1.49, 6.57]) and (adjusted, OR 1.81; 95% CI [1.19, 2.74]), respectively (Figures I and II in the Supporting Information). In an additional analysis, which only included results from interviews with the patient alone, calling EMS and high patient‐perceived symptom severity were still associated with hospital arrival within 180 min (Figures III–VI in the Supporting Information).

## 4 DISCUSSION

In this cross‐sectional, two‐center survey, we explored the importance of patient‐reported factors for early hospital arrival within 180 min from stroke symptom onset. Notably, we found that patients or bystanders choosing EMS as the first choice of contact for medical assistance and patient‐perceived high symptom severity were significantly associated with hospital arrival within 180 min of symptom onset, while living alone was associated with a decreased likelihood of arrival within 180 min.

Interestingly, 46.3% of patients arrived to a hospital within 180 min and 53.0% within 270 min. These rates are slightly above those reported in the Danish National Stroke Registry 2018 (Damgaard & Vögele, 2019), in which 40% and 49% of the patients arrived within 180 and 270 min, respectively (Johnsen et al., 2016). These arrival times correspond to those reported 10 years ago, when 46% of patients with TIA and minor stroke sought medical attention within the first 3 h of symptom onset (Chandratheva et al., 2010). This stagnation in overall patient response times highlights the need to address the patients’ persistent reluctance to call for immediate help. The choice of calling EMS as the first contact for medical help is essential for activation of the stroke chain of survival that includes rapid dispatch of an acute response ambulance and arrival to a stroke unit. In our study, patient characteristics relevant to contacting EMS within 180 min of symptom onset were the presence of prior stroke or current severe stroke symptoms. Our finding supports previous studies, which report that calling EMS as the first contact is a strong predictor for early arrival (Agyeman et al., 2006; Ekundayo et al., 2013; Morris et al., 2000; Puolakka et al., 2016; Rossnagel et al., 2004; Soomann et al., 2015; Soto‐Camara et al., 2019). In contrast, calling the patient's GP as the first contact was significantly associated with longer prehospital delays (Faiz et al., 2017; Fladt et al., 2019). A study including 299 patients (median age 75, 48.5% females) with acute ischemic stroke (*n* = 254) and intracerebral hemorrhage (*n* = 47) reported that 61 patients contacted the GP as the first contact, and of these, 37 patients were asked to see the GP in person instead of calling EMS (Faiz et al., 2017). Many patients who visited the GP office had significantly lower National Institutes of Health Stroke Scale (NIHSS) scores, significantly longer the first contact and prehospital delays, and significantly lower rate of thrombolysis treatment compared with those who called EMS first (Faiz et al., 2017). In a study of 336 patients (median age 74, 40% women) with ischemic stroke, one in three patients who first called the GP, followed by a face‐to‐face visit, had the odds for prehospital delay quadrupled compared to those who called the GP (Fladt et al., 2019). Patients who arrived later that 270 min after stroke symptom onset were also more likely to live alone and have a significantly lower NIHSS score (Fladt et al., 2019). The first contact to GP, followed by a face‐to‐face visit, added significantly to prehospital delays in both studies and could be a factor to address in future stroke awareness campaigns for GPs. In our study, when patients chose to call their GP, the median time from stroke symptom onset to the first contact was 11‐fold higher than those who called EMS, with the major time delay occurring between symptom onset and the decision to seek medical advice (Figure 4).

A study including 322 patients with ischemic stroke found that 19.6% of the patients perceived their symptoms as serious (the highest level), which was associated with hospital arrival within 210 min (Soto‐Camara et al., 2019). Similarly, of 149 patients (mean age 69.3, 40% women) presenting with ischemic stroke, only 11% perceived their symptoms as extremely serious (the highest level), which was associated with a hospital arrival within 210 min (Mellon et al., 2016). Another study of a mixed stroke population (diagnosis codes I61, I63, and G45) with 384 stroke patients and 264 bystanders found that the patient or bystander perception situation was very serious (the highest level), was associated with primary EMS contact and hospital arrival within 180 min (Iversen et al., 2020). Having a bystander who perceives the situation as very serious at stroke symptom onset increased the likelihood of revascularization therapy, as their perception prompted a primary call to EMS (Iversen et al., 2020). However, if the bystander did not perceive the situation as very serious, the benefit of having a bystander present at stroke onset was negated. Interestingly, the call to EMS was made by the patient in 122 cases (55.0%) and by the bystander in 59 cases (26.6%). Including bystanders as an important factor seems essential as stroke recognitions by a bystander was significantly higher for those patients who arrived early in our study (Table 1). In the current study, the level of perceived severity was assessed using a numeric rating scale (0–100), with 100 being the most severe (Streiner and Norman, 2008). Any definition of high patient‐perceived severity is relative to previous experience and coping strategies for the individual (Streiner and Norman, 2008). We pragmatically defined the high patient‐perceived severity level using the median ≥25 as the cutoff. Of note is the low median, indicating that the majority of patients may not consider stroke as a medical emergency.

Previous knowledge of one or more typical stroke symptoms or revascularization treatment before hospital admission was not significantly associated with early arrival in our study. Interviews in an earlier study revealed that patients expected symptoms of a new stroke to be similar to their previous symptoms (Amtoft et al., 20212021). At the time of inclusion in the present study, no nationwide public stroke awareness campaign had been launched in Denmark, which could influence stroke knowledge and/or behavioral responses in our population to stroke symptoms, as well as contribute to the almost equal proportions of patients first calling EMS (*n* = 147) versus their GP (*n* = 140) observed in our study. The choice of not calling EMS could, however, also be due to the objectively mild stroke severity of the included patients, the patients’ perception of the symptom as nonsevere, or that participants did not consider the situation as a medical emergency. We saw no difference in symptom presentation according to typical symptoms versus atypical symptoms when contacting EMS or the GP (31.3% vs. 29.2%, respectively).

Mass media interventions have been implemented to improve stroke recognition and emergency response (American Stroke Association, 2018). Such campaigns are based on the premise that stroke symptom awareness is associated with immediate activation of EMS. However, the effect of these campaigns on early arrival have been inconclusive. Populations may show an increase in stroke knowledge but they showed no changes in their behavior or decision making during stroke symptom onset, and the proportion of patients arriving early did not increase as expected (Fassbender et al., 2013; Lecouturier et al., 2010; Teuschl & Brainin, 2010). In contrast, the Swedish National Stroke Campaign was associated with significant increases in the proportion of stroke patients who arrived at a hospital within 180 min and in the number of patients receiving revascularizations therapy up to 1 year after the end of the campaign. The population included 97,840 patients registered in the Swedish Stroke Registry who were diagnosed with ischemic stroke, intracerebral hemorrhage stroke, or unspecified stroke (Nordanstig et al., 2019). Diagnosis of TIA was not included, which may explain why the proportion receiving revascularization therapy increased. Acknowledgment of symptoms and fast response may be affected by stroke location in the brain, as right hemisphere stroke may induce a reduced symptom awareness (stroke‐induced anosognosia). We found no association between response to symptoms and stroke location; early arrival was equally frequent in patients with right or left hemisphere injuries, as has been previously reported (Fladt et al., 2019).

### 4.1 Limitations

To reduce recall bias, we enrolled patients as early as possible after stroke onset. Though we aimed to consecutively include patients, some patients were discharged before enrollment was possible. Moreover, some patients died or were admitted to intensive care units, which may represent selection bias. These scenarios could have reduced the number of included patients with either very high or very low symptom severity.

## 5 CONCLUSION

In a mixed stroke population, an increased perception of symptom severity and choice of the first contact to EMS, rather than to the patient's GP, increased the likelihood of hospital arrival within 180 min after stroke symptom onset. These findings suggest that behavioral motivators and barriers related to quickly contacting EMS, either by the patient or a bystander, upon symptom onset and recognizing stroke symptom severity, need to be further addressed.

## CONFLICT OF INTEREST

The authors declare that there is no conflict of interest.

### PEER REVIEW

The peer review history for this article is available at https://publons.com/publon/10.1002/brb3.2225.

## Supporting information

SuppMat1

SuppMat2

SuppMat3

SuppMat4

SuppMat5

SuppMat6

SuppMat7

SuppMat8

SuppMat9
