# Supplementary figures and images for: Patient‐reported factors associated with early arrival for stroke treatment
Source: Brain Behav. 2021 Jun 4;11(8):e2225. doi: 10.1002/brb3.2225 (PMC8413799; doi:10.1002/brb3.2225)

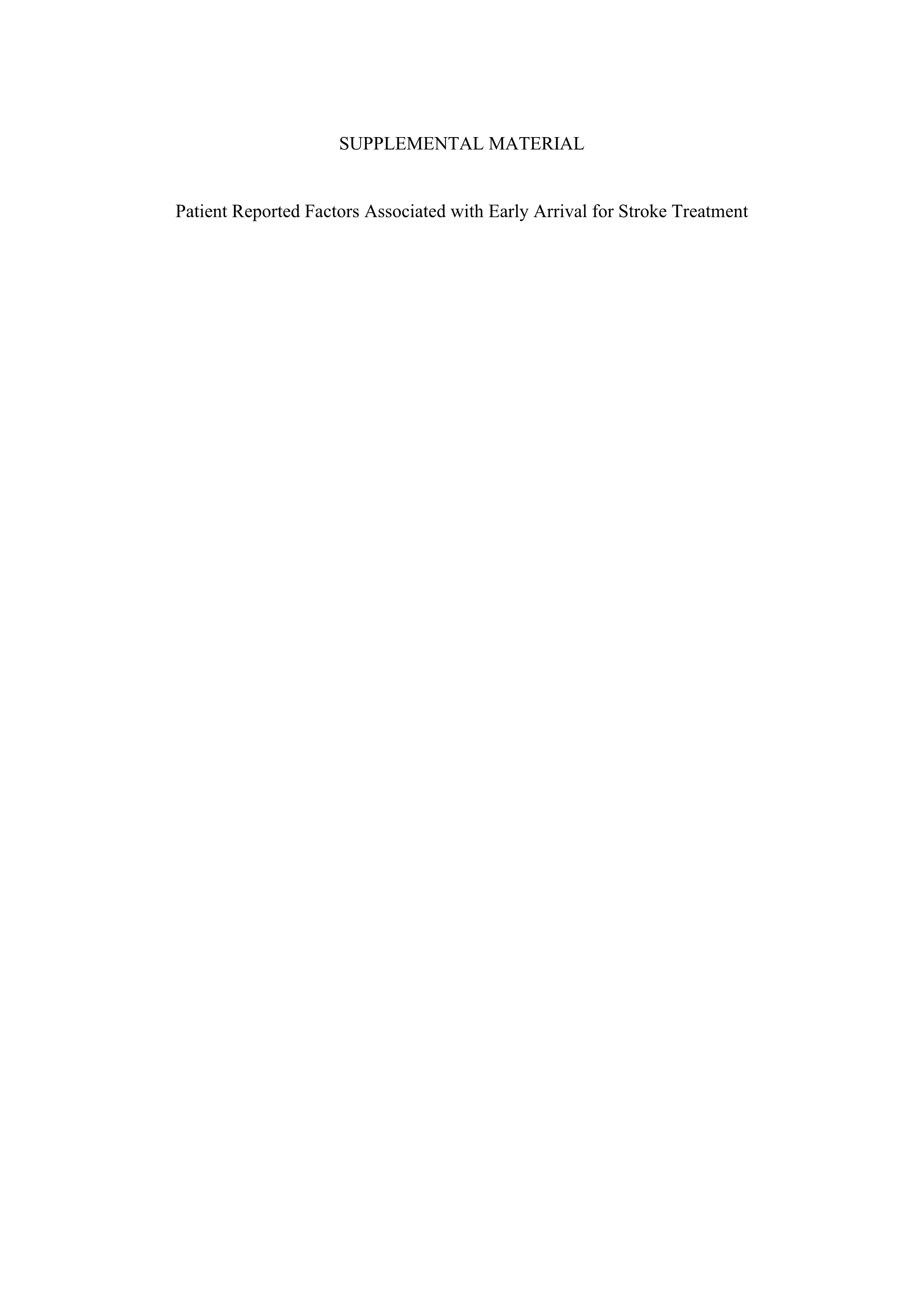

Supplement: Supplementary file 1 — SuppMat1 [file BRB3-11-e2225-s004.jpg]

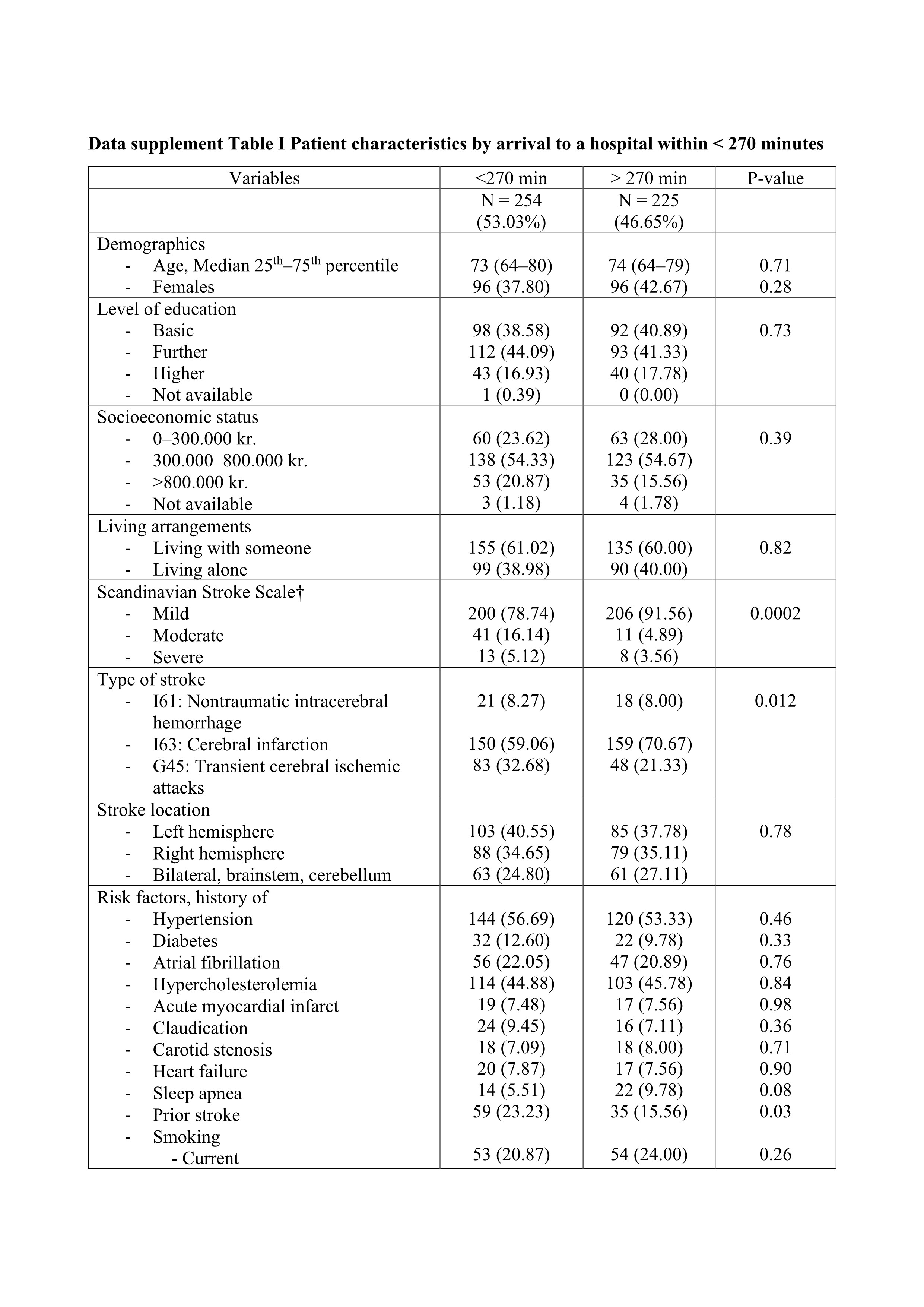

Supplement: Supplementary file 2 — SuppMat2 [file BRB3-11-e2225-s006.jpg]

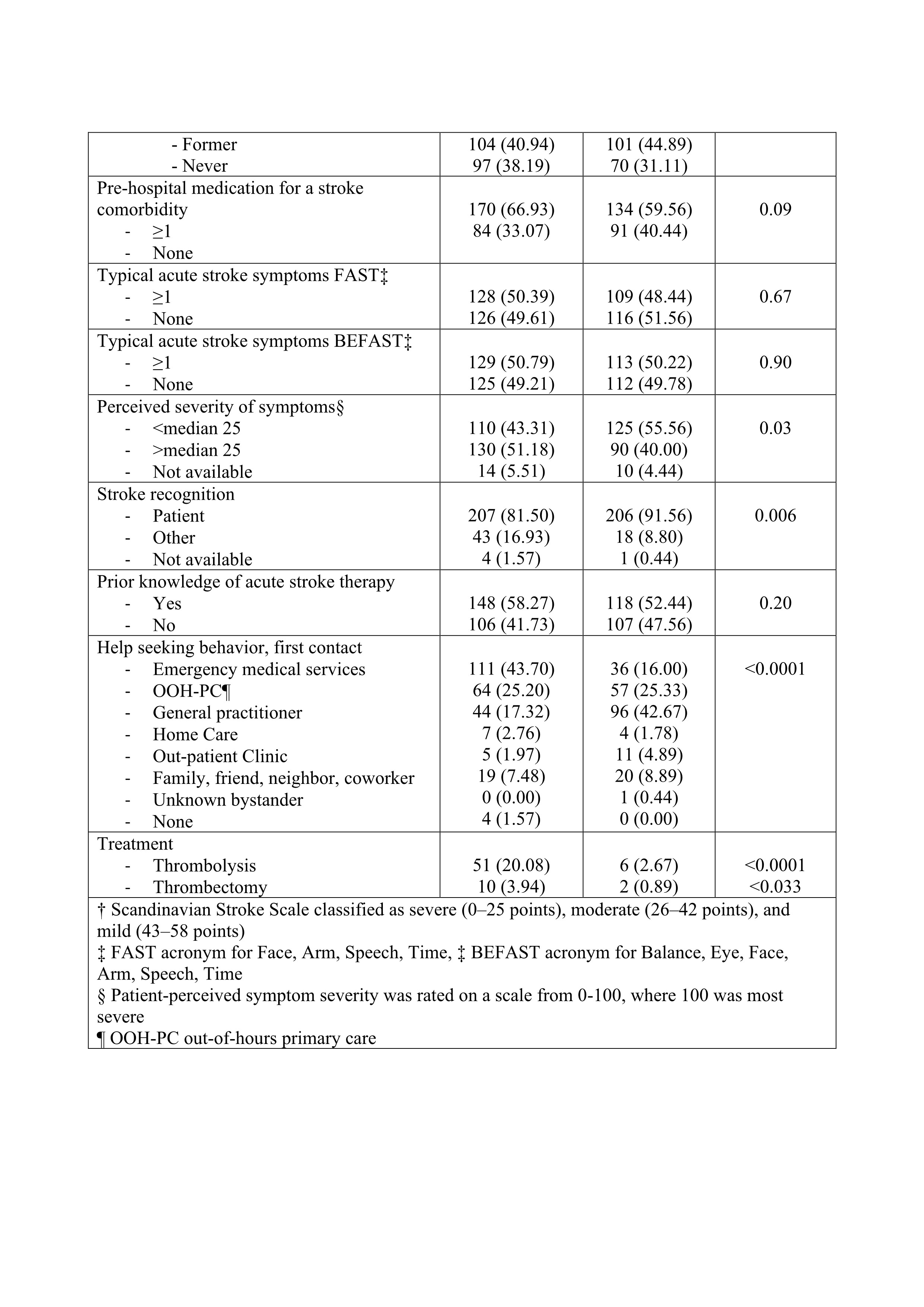

Supplement: Supplementary file 3 — SuppMat3 [file BRB3-11-e2225-s001.jpg]

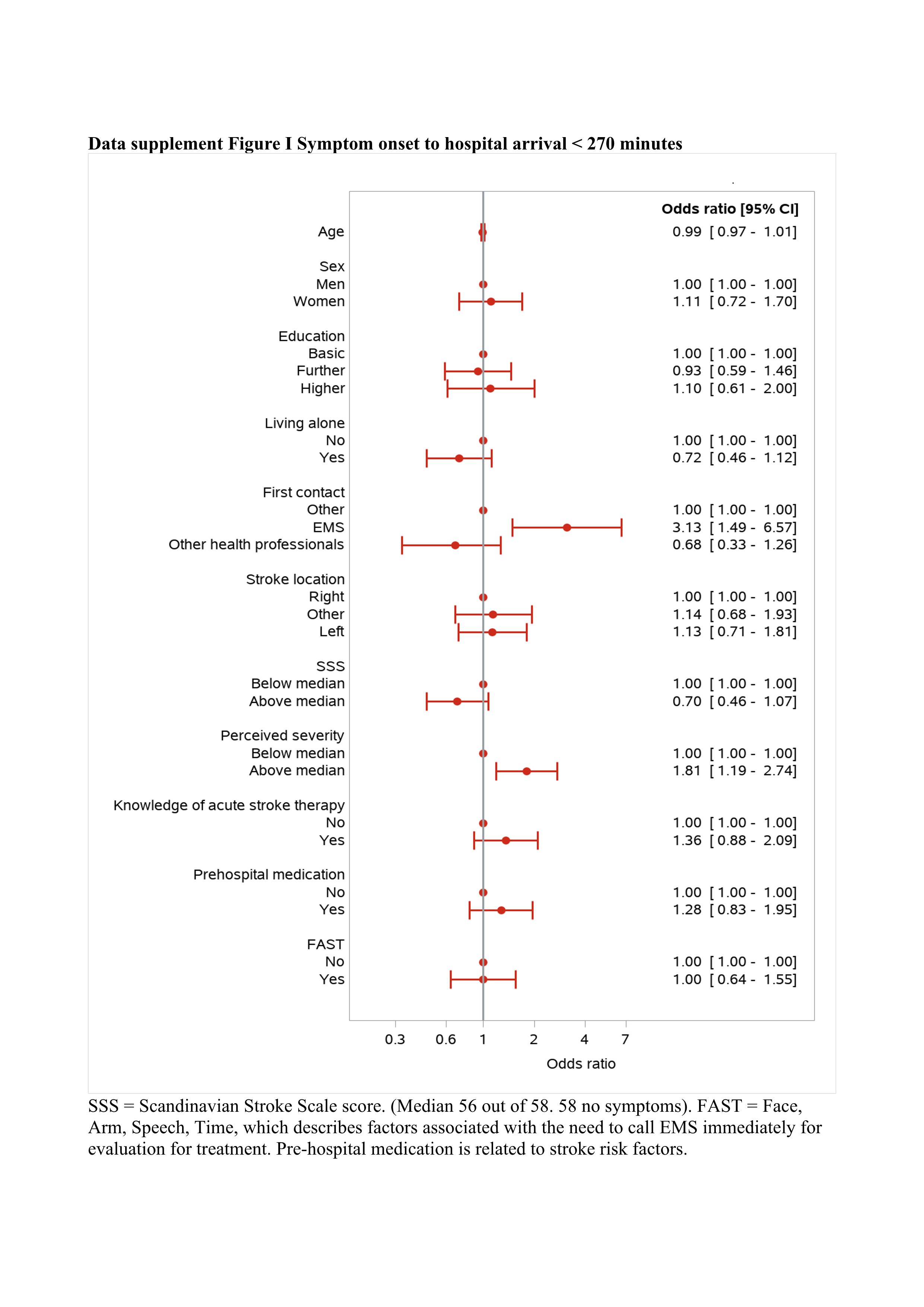

Supplement: Supplementary file 4 — SuppMat4 [file BRB3-11-e2225-s003.jpg]

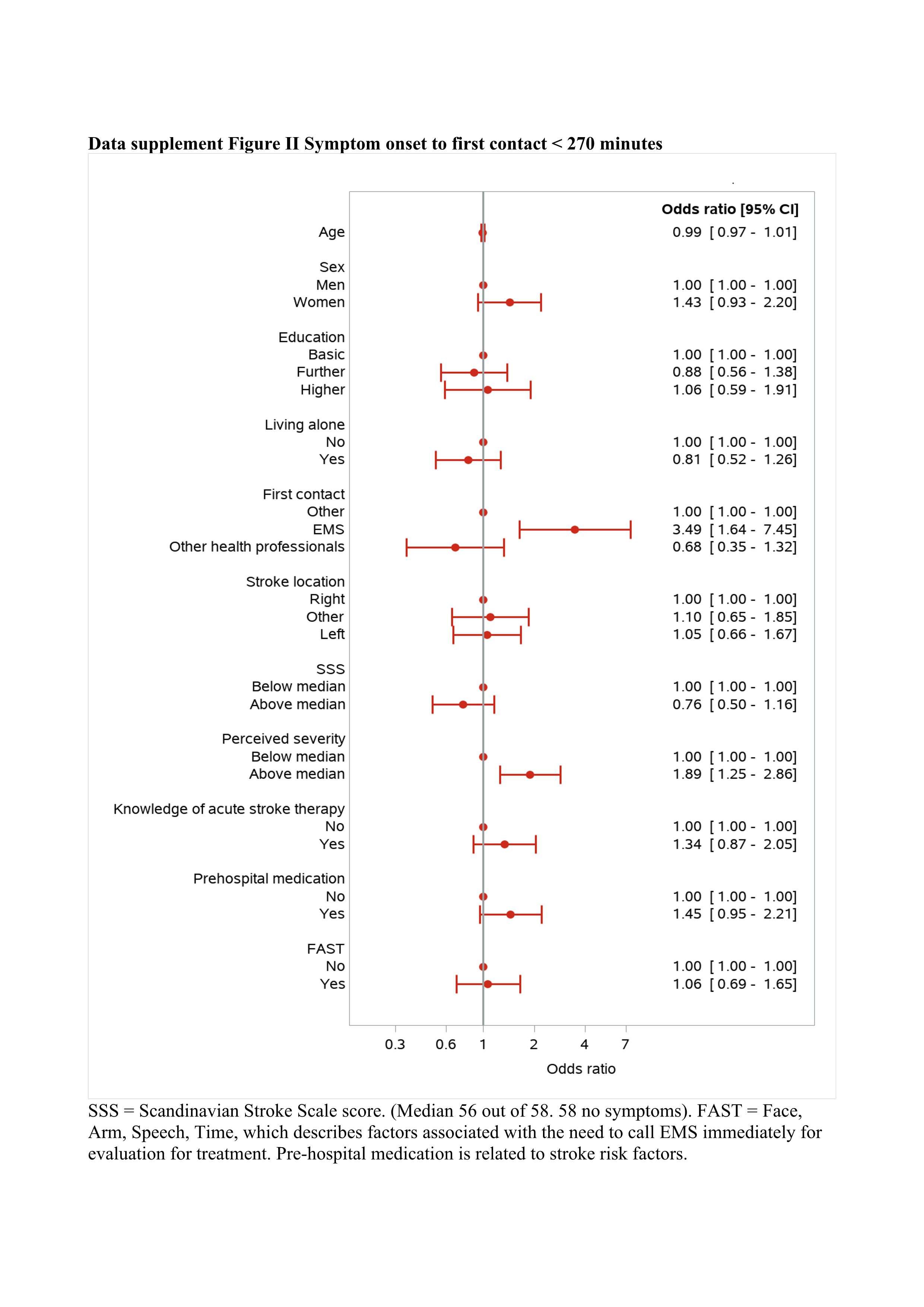

Supplement: Supplementary file 5 — SuppMat5 [file BRB3-11-e2225-s008.jpg]

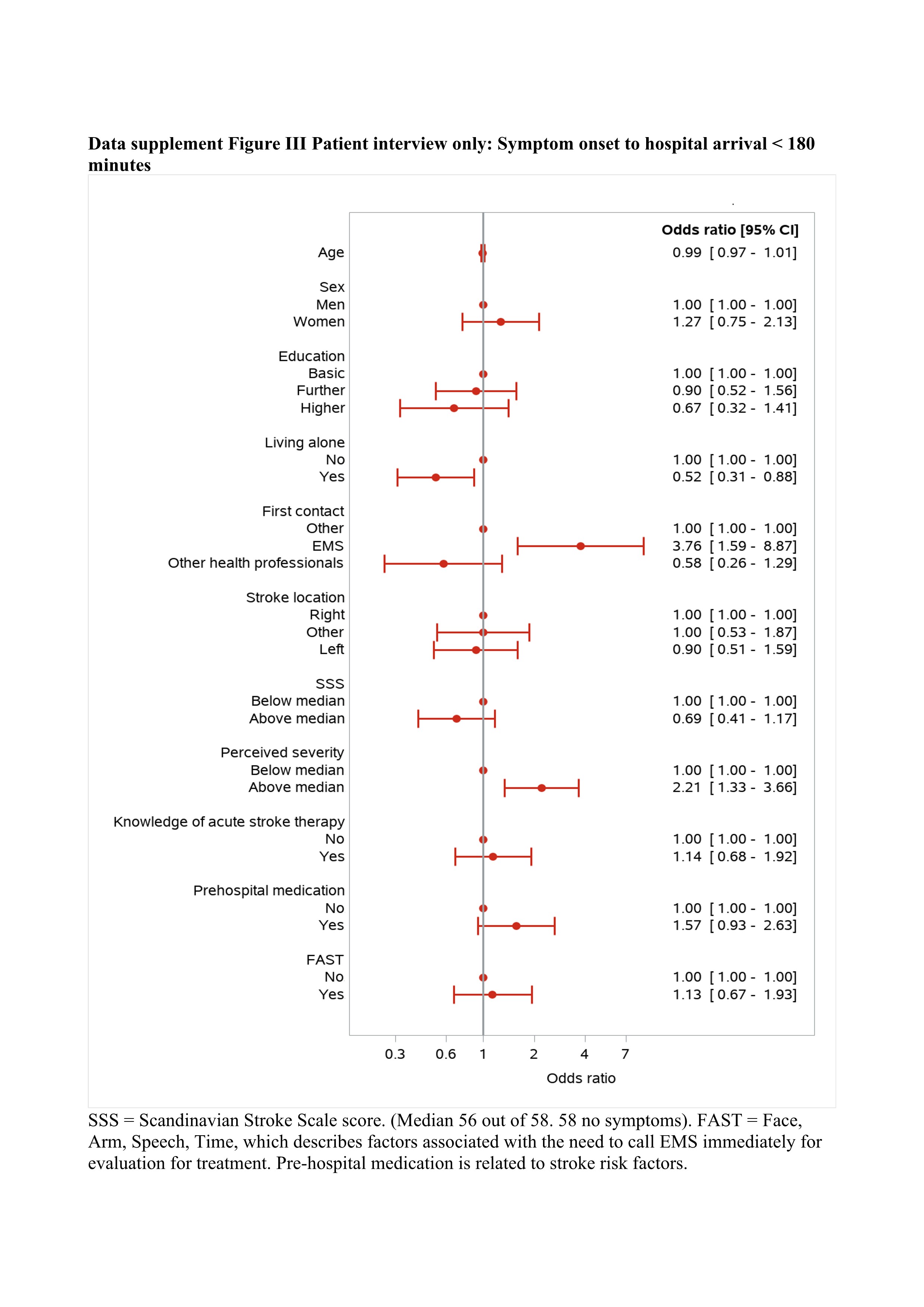

Supplement: Supplementary file 6 — SuppMat6 [file BRB3-11-e2225-s007.jpg]

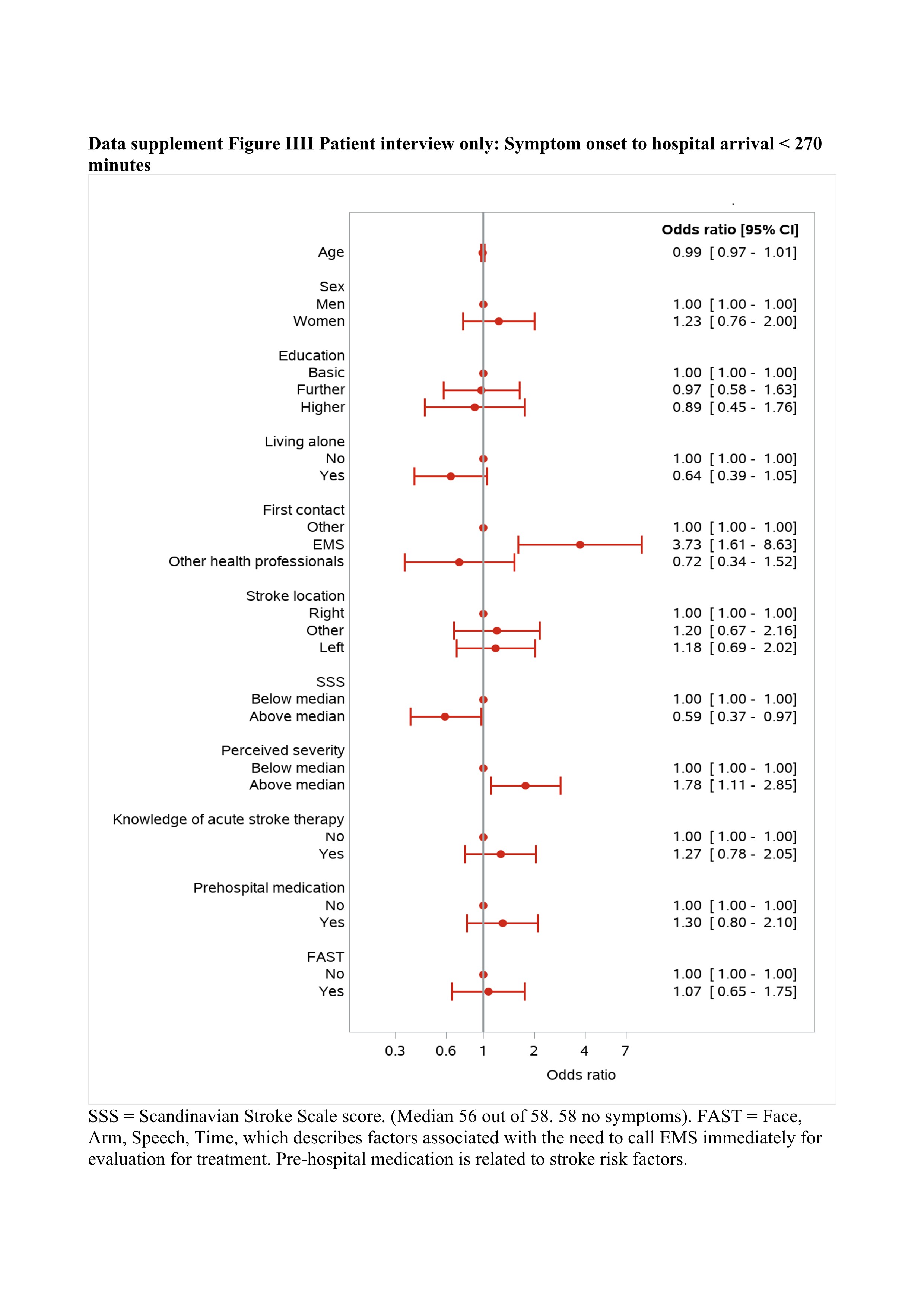

Supplement: Supplementary file 7 — SuppMat7 [file BRB3-11-e2225-s009.jpg]

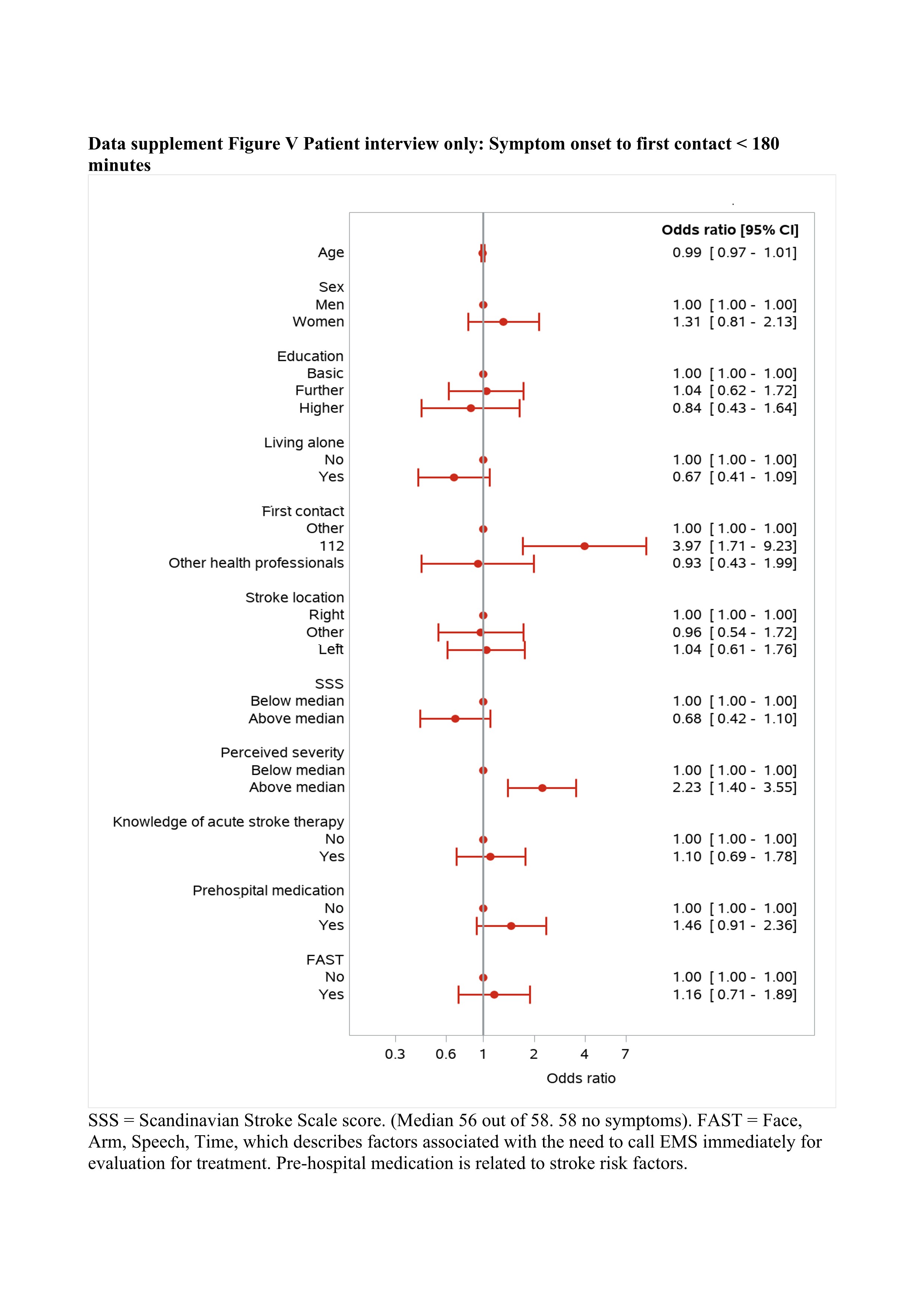

Supplement: Supplementary file 8 — SuppMat8 [file BRB3-11-e2225-s002.jpg]

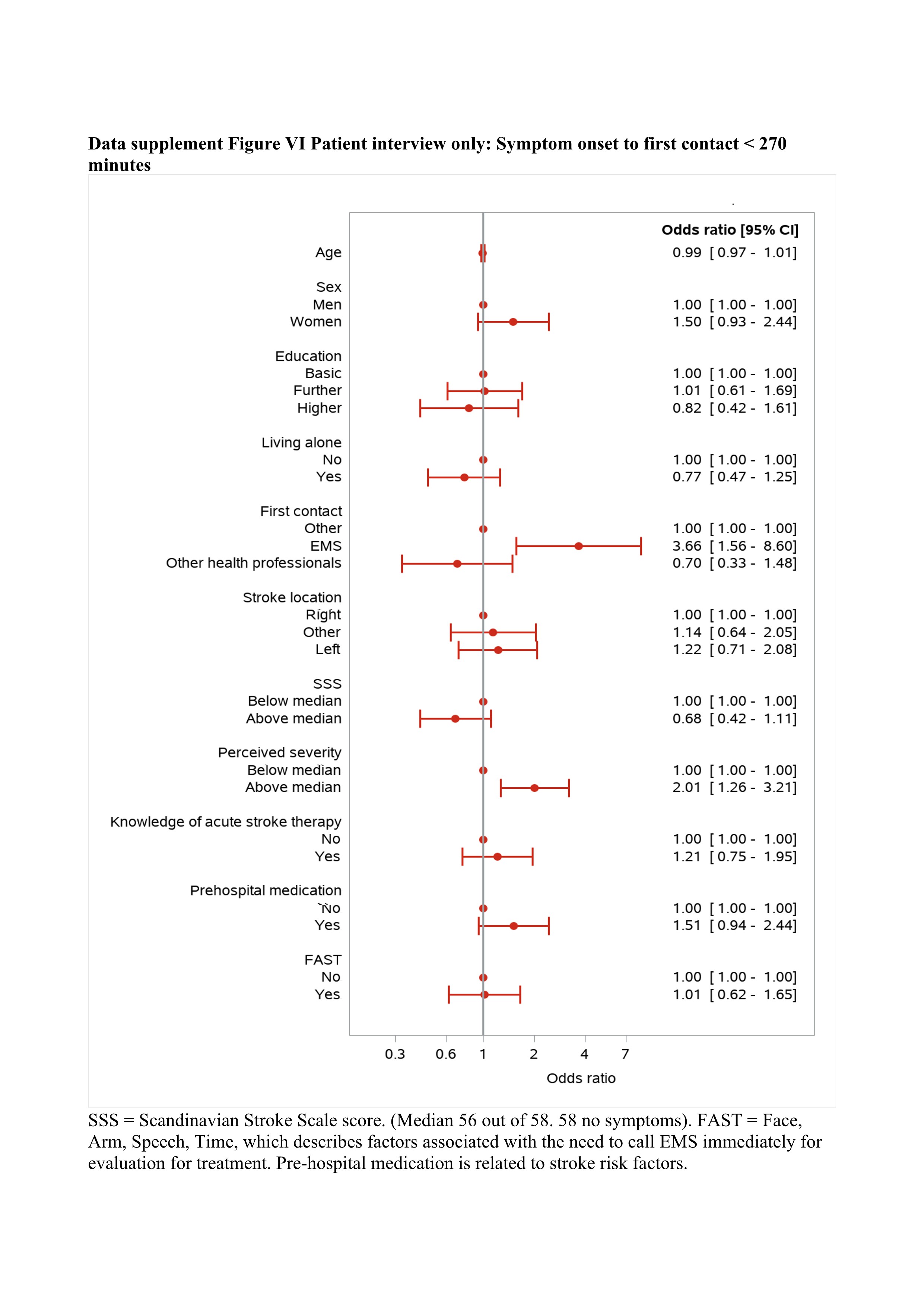

Supplement: Supplementary file 9 — SuppMat9 [file BRB3-11-e2225-s005.jpg]
